# The gap between self-reported and medically confirmed Gender Incongruence/Gender Dysphoria among students in china

**DOI:** 10.1192/j.eurpsy.2022.840

**Published:** 2022-09-01

**Authors:** Y. He, Y. Pan, X. Chen

**Affiliations:** 1 The Second Xiangya Hospital of Central South University, Department Of Psychiatry, Changsha, China; 2 University of Bath, Department Of Psychology, Bath, United Kingdom

**Keywords:** Gender Incongruence, Gender Dysphoria, Transgender

## Abstract

**Introduction:**

As the incidence of gender incongruence (GI)/gender dysphoria (GD) rises yearly, public understanding of transgender is also increasing, whereas this improvement cannot be achieved without extensive transgender-related surveys. However, most of the surveys were only issued to people who identify themselves as transgender with the absence of medical confirmations in most situations. These result in a gap between transgender survey and diagnosed GI/GD.

**Objectives:**

This study aims to discover the gap between self-reported and diagnostically confirmed transgender and GI/GD individuals among students in China.

**Methods:**

We chose two middle schools and one college from Changsha (a city in China) at random with a total of 2047 students. Among them, 1661 students gave us certain gender identify responses in which we categorized them into two types (cisgender and gender minorities). Professional psychiatrists then used ICD-11 and DSM-5 criteria to confirm whether the self-reported gender minorities could be diagnosed with GI/GD via phone or in person.

**Results:**

In total, 7.5% of the college students and 5.8% of the middle school students reported themselves as gender minorities. Although 29% of college students and 43.8% of middle school students did not cooperate with the subsequent psychiatric interviews, none of the self-reported gender minority students meet the GI/GD criteria of ICD-11/DSM-5.

**Conclusions:**

The epidemiological investigation of transgender is heavily affected by the definition and the data sources. There is a huge heterogeneity between self-reported transgender and diagnosed GI/GD. Future transgender studies should strictly control inclusion criteria.

**Disclosure:**

No significant relationships.

